# ﻿Taxonomic review of the genus *Clethrorasa* Hampson, 1908 (Lepidoptera, Noctuidae, Amphipyrinae), with descriptions of a new genus and a new species from southeastern China

**DOI:** 10.3897/zookeys.1248.145465

**Published:** 2025-08-08

**Authors:** Yue Qin, Jiang Zhu, Huilin Han

**Affiliations:** 1 School of Forestry, Northeast Forestry University, Harbin 150040, China Northeast Forestry University Harbin China; 2 State Environmental Protection Key Laboratory of Urban Ecological Simulation and Protection, South China Institute of Environmental Sciences, MEE, Guangzhou 510530, China South China Institute of Environmental Sciences Guangzhou China; 3 Northeast Asia Biodiversity Research Center, Northeast Forestry University, Harbin 150040, China Northeast Forestry University Harbin China; 4 Ministry of Education, Key Laboratory of Sustainable Forest Ecosystem Management, Northeast Forestry University, Harbin 150040, China South China Institute of Environmental Sciences Guangzhou China

**Keywords:** *Clethrorasa*, key, new genus, new species, new synonymy, southeastern China, taxonomic revision

## Abstract

In this study, the moth genus *Clethrorasa* Hampson, 1908 is reviewed, and a new genus, *Minclethrorasa***gen. nov.** with its type species, *M.chinensis***sp. nov.** are described from southeastern China. A new synonymy is established for the species *Clethrorasatibetica* Orhant, 2013 = *Clethrorasakossnerae* Behounek, 1997, **syn. nov.** Additionally, *Clethrorasapilcheri* (Hampson, 1896) is reported for the first time from China. The adult and genitalia characteristics of all species are illustrated, and a key is provided for identifying species of the two genera.

## ﻿Introduction

The genus *Clethrorasa* Hampson, 1908 is a group of noctuids with a distinctive black and white appearance. It was established by Hampson based on *Leocymapilcheri* Hampson, 1896 as the type species. Until now, four species have been described in this genus, all distributed in the Oriental Region: *C.pilcheri* Hampson, *C.micropuncta* Holloway, *C.kossnerae* Behounek and *C.tibetica* Orhant. The taxonomic status of *Clethrorasa* is still debated: originally placed in the subfamily Acronyctinae (=Acronictinae) (Hampson 1908, 1910), but later classified as the subfamily of Amphipyrinae (Poole 1989; Holloway 1989; Yoshimoto 1995; Behounek 1997; Chen 1999; Fibiger and Hacker 2007; Kononenko and Pinratana 2013; Han and Yao 2018; Gielis et al. 2022). Some scholars have differing opinions on its taxonomic status (Holloway 2011; Kononenko and Pinratana 2013; Wang et al. 2018). The genus was first mentioned in the key compiled by Hampson (1908) but was not officially described until 1910. Its type species, *C.pilcheri* (Hampson, 1896), was originally recorded in the northeastern Himalayas (Sikkim, India). Holloway (1976, 1989) recorded the species from the Malay Archipelago (Borneo), while Kononenko and Pinratana (2013) reported it from mainland Southeast Asia (Thailand). Holloway (1989) described a second species: *C.micropuncta* from Ulu Temburong in Brunei. A third species, *C.kossnerae*, was described by Behounek in 1997 from northern Vietnam (Cha-pa, Prov. Lào Cai) and southern China (Prov. Guangdong) (Behounek 1997). In 2013, *C.tibetica* was described from the Xizang Autonomous Region (= Tibet) in China, becoming the fourth species of the genus (Orhant 2013).

Based on specimens collected from southern China and Borneo (Malaysia), we have systematically examined the genus *Clethrorasa* through morphological comparisons, and we identified a new species of a new genus from southeastern China (Jiangxi, Fujian, Guangdong Provinces, and Guangxi Zhuang Autonomous Region): *Minclethrorasachinensis* gen. et sp. nov. It was observed that there are no significant differences in appearance or genitalia morphology between *C.tibetica* and *C.kossnerae*. Therefore, we consider *C.tibetica* to be a synonym of *C.kossnerae*. Additionally, illustrations of the adults and genitalia of all the mentioned species, a worldwide distribution map, and a key for the genus are provided.

## ﻿Material and methods

This study is based on specimens in the collection of the Insect Taxonomy Laboratory in the Northeast Forestry University, which includes moth specimens collected using high-pressure mercury lamps or black light lamps in southern China and Borneo in recent years. The method for preparing genitalia slides follows the protocol established by Kononenko and Han (2007). Adult specimens and genitalia slides were photographed using a Nikon D700 camera and an Olympus photo microscope. The images were then imported into Helicon Focus v. 7.6.6 software for depth stacking. A distribution map was generated using QGIS v. 3.38.2. The processed images were edited with Adobe Photoshop 2020.21.0.0 and compiled into a plate. The research specimens, including type materials of the new species, are deposited at the Insect Taxonomy Laboratory of Northeast Forestry University (**NEFU**).

## ﻿Taxonomic account

### ﻿Genus Minclethrorasa gen. nov.

Taxon classificationAnimaliaLepidopteraNoctuidae

FECFB2C6-3A31-59B0-AA1E-C8118EAC37B1

https://zoobank.org/6E747967-BEF6-4FD8-9F32-3110BF6F360B

#### Type species.

*Minclethrorasachinensis* sp. nov.

#### Diagnosis.

The new genus is closely related to *Clethrorasa* in coloration and wing shape but can be unambiguously distinguished by both external morphology and genitalic structures, as comprehensively detailed in Table 1.

**Table 1.** Comparison of the characteristics of genera *Minclethrorasa* and *Clethrorasa*.

Characteristics		Minclethrorasa	Clethrorasa
**external morphology**	wingspan	24–30 mm	29.0–38.5 mm
	abdomen	color off-white	color mostly black
	forewings	black spots small, but numerous	black spots large, but few
	hindwings	light in color, slightly darker on the outer 1/4	dark in color, often have white patches in males
**male genitalia**	uncus	hook-shaped	sickle-shaped
	tegumen	helmet-shaped	tongue-shaped
	valva	narrow, not very developed	broad, well developed
	saccus	U-shaped	V-shaped
	juxta	bullhead-shaped	claw-shaped
	vesica	with multiple medial diverticula	without medial diverticula
**female genitalia**	papillae anales	petal-shaped	nail-shaped
	ductus bursae	membranous	partly sclerotized
	corpus bursae	long pouch-shaped, with a signum	tie-shaped, without signa

#### Description.

**Adult.** Wingspan 24.0–30.0 mm. The color of head, dorsal side of thorax, patagia, tegulae, and forewings generally white. Antennae black-brown and linear. Proboscis well developed, with the labial palpus extending upward. Forehead smooth and devoid of protrusions. Compound eyes large. Two pairs of prominent black spots located in disc area of thorax, patagia with pair of black spots; each side of metathorax displaying a cluster of radiating white hairs. Abdomen off-white; several segments of abdominal base adorned with brushes on dorsal side; anal tuft white, extending straight out from the posterior end of abdomen; tufts of hair arranged on lateral sides of abdomen white at distal half. Legs partially white, with white ring at each segment on the base of tarsus. Forewings slightly narrow, scattered with black, metallic blue glossy dots, blocks, or stripes. Hindwings broad, mostly off-white, with light brown outer 1/4. ***Male genitalia*.** Uncus flattened, hook-shaped; tegumen broad, dorsal lobe covered in granules; subscaphium droplet-shaped; valva fairly regular, with slight protrusions and absent harpe; vinculum slender; saccus U-shaped; aedeagus cylindrical, featuring a well-developed vesica with multiple medial diverticula and moderately abundant cornuti. ***Female genitalia*.** Papillae anales petal-shaped; anterior and posterior apophysis processes relatively slender; ductus bursae membranous, short and simple; corpus bursae long, pouch-shaped, with longitudinal folds and a strong sclerotized signa plate.

#### Remarks.

The forewings of *Minclethrorasa* are relatively narrow and elongate, with rounded apices and anal angles, and the coloration of the head, thorax, and forewings is similar to that of the genus *Clethrorasa*, but the body shape and genitalia differ markedly from those of all known species of *Clethrorasa*. Therefore, *Minclethrorasa* is treated here as a new genus.

#### Distribution.

China (Fig. 22).

#### Etymology.

The new genus is named *Minclethrorasa*, formed by combining the generic name *Clethrorasa* with the prefix min- (derived from Latin ‘*minus*’, meaning ‘smaller’). This nomenclature reflects its morphological similarity to *Clethrorasa* while emphasizing its diminutive body size.

### ﻿ Minclethrorasa chinensis sp. nov.

Taxon classificationAnimaliaLepidopteraNoctuidae

E93E9440-73AD-5598-AD5C-F466D3E03F23

https://zoobank.org/FB4384D0-AC73-4BC8-985A-D5726FB4499E

**Figures 1–8.**
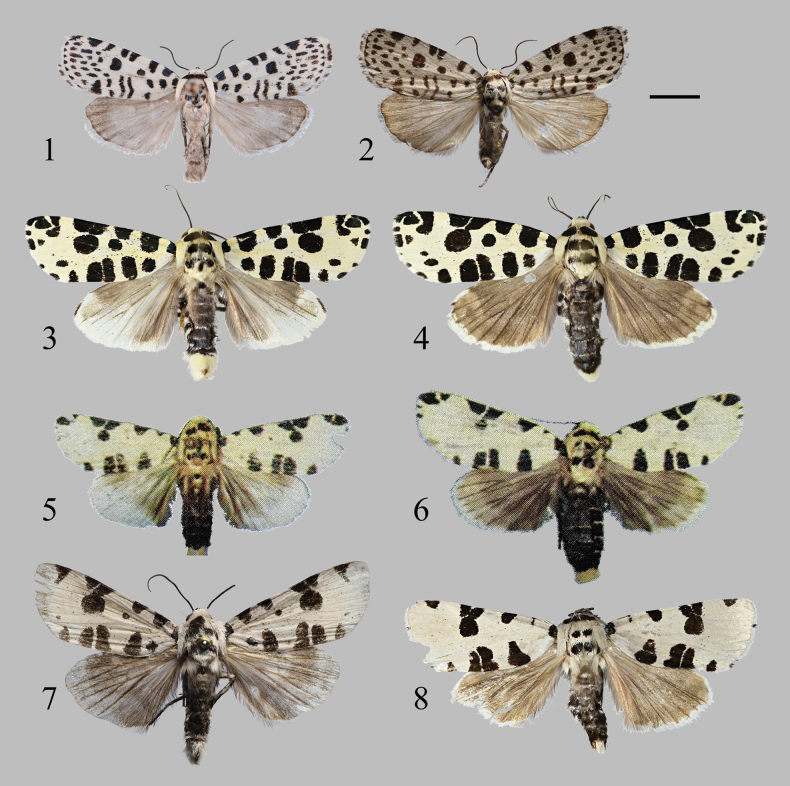
Adults of *Minclethrorasa* and *Clethrorasa* spp. (1, 3, 5, 7: male, 2, 4, 6, 8: female): 1. *M.chinensis* gen. et sp. nov., holotype, Guangdong, China (NEFU); 2. Ditto, paratype, Jiangxi, China (NEFU); 3, 4. *C.kossnerae* Behounek, 1997; 5, 6. *C.micropuncta* Holloway, 1989 (after Holloway 1989); 7, 8. *C.pilcheri* (Hampson, 1896). Scale bar: 5mm.

**Figures 9–16.**
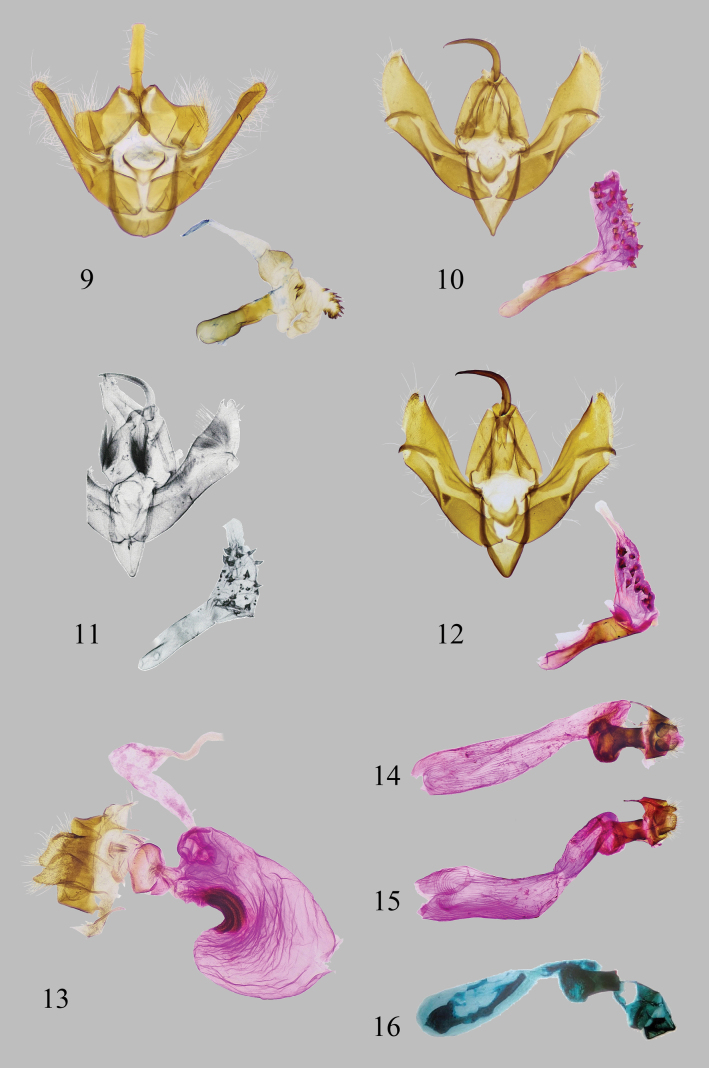
Genitalia of *Minclethrorasa* and *Clethrorasa* spp. (9–12: male, 13–16: female): 9, 13. *M.chinensis* gen. et sp. nov.; 10, 14. *C.kossnerae* Behounek, 1997; 11. *C.micropuncta* Holloway, 1989 (after Holloway 1989); 12, 15. *C.pilcheri* (Hampson, 1896); 16. *C.tibetica* Orhant, 2013 (after Orhant 2013).

Figs 1 , 2 , 9 , 13 , 22 , 23 Chinese common name 小飘夜蛾 (Tiny Fluttering Noctuid)

#### Type material.

***Holotype***: • male, China, Guangdong, Heyuan, Zijin, Baixi Reserve, 16.III.2023, leg. Y. Wu and J. Zhu, genit. prep. HHL-5798-1. ***Paratypes***: • 1 female, China, Jiangxi, Ganzhou, Longnan, Jiulianshan Nature Reserve, Hengkengshui Protection Station, 24–25.VII.2021, leg. T.T. Zhao et al., genit. prep. HHL-7046-2; • 2 males, China, Jiangxi, Ganzhou, Longnan, Jiulianshan Nature Reserve, Zhiqing Hotel, 16–17.VII.2021, leg. T.T. Zhao et al., genit. prep. HHL-7047-1 and 7048-1; • 1 male, China, Guangxi, Longsheng, Huaping National Reserve, 10–14.VII.2021, leg J.J. Fan and B. Gao, genit. prep. HHL-7049-11; • 1 male, China, Jiangxi, Ganzhou, Jiulianshan Nature Reserve, Daqiutian Protection Station, 18–20.VII.2021, T.T. Zhao, genit. prep. HHL-7050-1; • 1 male, China, Guangdong, Shaoguan, Chebaling, 29.IV–3.V.2019, leg. H.L. Han et al., genit. prep. HHL-7051-1; • 1 male, 1 female, China, Fujian, Wanmulin Nature Reserve, V.1986, unknown collector, genit. prep. HHL-7052-1 and 7053-2.

#### Diagnosis.

This species closely resembles the three species in the genus *Clethrorasa*, but it can be distinguished based on the following characteristics (these characteristics are highly similar among *Clethrorasa* spp.): In terms of external morphology, this species exhibits a smaller body size (wingspan 24–30 mm vs 29.0–38.5 mm), with mostly grayish-white forewings and relatively small but numerous black spots. The veins R and M1 of the hindwings are relatively short, close to 1/3 of the hindwing length (Fig. 23). The dorsal surface of abdomen is off-white instead of mostly black. In male genitalia, the uncus is straight and shaped like a ball rod rather than a sickle; the tegumen is helmet-shaped rather than tongue-shaped; the valva is relatively narrow and not very developed; the saccus is in a ‘’U’’ shape instead of a ‘’V’’ shape, with a smooth bottom; the juxta is shaped like a bull’s head instead of a claw; the aedeagus is relatively short and thick, with a well-developed vesica with multiple medial diverticula; the cornuti more slender and clustered in two distinct patches. In female genitalia, the papillae anales are relatively wide, the ductus bursae is relatively unsclerotized, the corpus bursae is relatively short, long pouch-shaped rather than tie-shaped, and has a strong, hardened spinal signum on the inner side.

**Figures 17–21.**
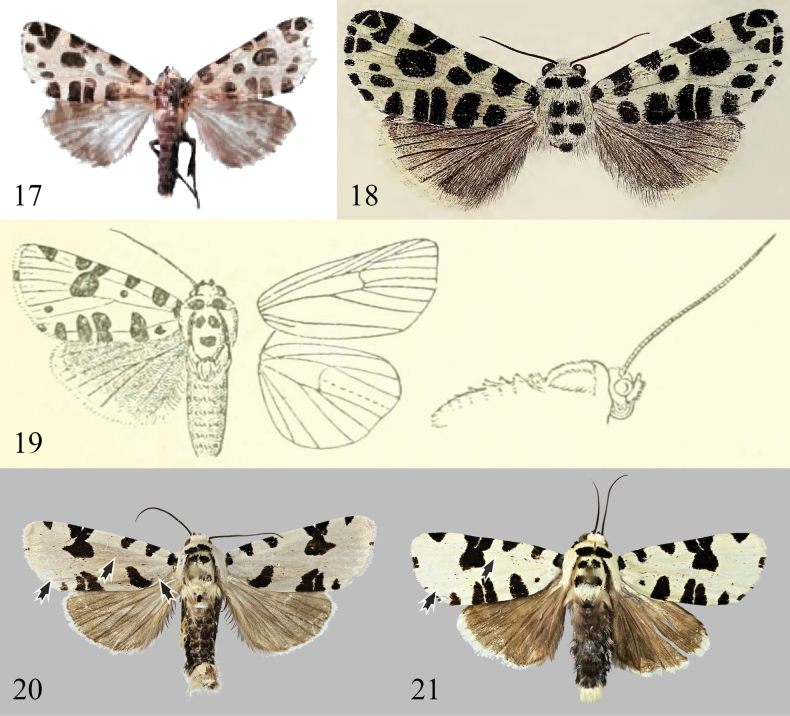
Adults of *Clethrorasa* spp.: 17. *C.tibetica* Orhant, 2013 (= *C.kossnerae* Behounek, 1997, syn. nov.), female, holotype (after Orhant 2013); 18. *C.kossnerae* Behounek, 1997, male, holotype (after Behounek 1997); 19. *C.pilcheri* (Hampson, 1896), male, type (after Hampson 1910); 20, 21. Ditto, males from Yunnan, China (20) and Sabah, Malaysia (21), the arrows indicate the intraspecific variation of spots of the species.

#### Description.

**Adult** (Figs 1, 2). Wingspan 24–30 mm. Head generally white; antennae blackish-brown. Dorsal side of thorax white, with four large purple-black spots; patagium white, with two prominent black spots located on the distal half; tegula white. Abdomen thin off-white, with a slight brownish tuft of hair at three terminal segments. Forewings white, with scattered black spots; transverse lines consisting of black spots, which are mostly shortband-shaped in costal margin area, the basal line only present as a black dot; antemedial line double, inner line formed by two black dots, exterior line by three black dots; median line double, formed by two black bands at inner and exterior lines, weakly incurved in inner margin area; postmedial line double, incurved posterior to end of cell, inner line formed by eight black dots or short bands, exterior line by seven black dots or short bands, the triangle spots at costal and inner margin areas are the largest; subterminal line parallel to postmedial line, formed by seven black dots; terminal line thin consisting of triangular black dots; orbicular spot medium, circular black dot; reniform spot large and black; fringe white with black patches. Hindwings pale-grayish with grayish-brown marginal shade darkest towards apex and costal and termen margins; discal spot indistinct; vein indistinct, brown.

***Male genitalia*** (Fig. 9). Uncus flattened, hook-shaped, apically nearly straight. Tegumen short and broad, shoulder helmet-shaped; dorsal lobe of tegumen covered in granules inside; subscaphium droplet-shaped; medial lobe of tegumen large, swollen. Valva sclerotized and narrow; costa shaped as a narrow strip, extending to cucullus, basal part incurved arc-shaped; sacculus short and irregular square-shaped; sacculus process narrow band-shaped, gradually widening apically, especially exceeding costa before cucullus; cucullus excurved arc-shaped. Juxta sclerotized, bullhead-shaped. Vinculum U-shaped; saccus broad tongue-shaped. Aedeagus cylindrical straight, gradually narrowing posteriorly; coecum short and coars; carina weakly sclerotized. Vesica membranous with three cornuti at middle part; diverticula short and smooth, nipple-shaped; subdiverticula bifurcated, one incurved and without cornuti, another short, covered with about eighteen cornuti; base of vesica ejaculatorius covered with thick graniculi, and rather expanded.

***Female genitalia*** (Fig. 13). Papillae anales petal-shaped, sclerotized. Apophyses anteriores and posteriores broad and approximately equal in length. Ostium bursae flat and straight. Ductus bursae membranous, curved, with oval-shaped swelling posteriorly. Corpus bursae long, pouch-shaped, curved towards one side in the middle, with a strong sclerotized signa plate in the middle of inner side. Appendix bursae produced at the posterior part of corpus bursae.

#### Distribution.

China (Jiangxi, Fujian, Guangdong, Guangxi).

#### Etymology.

The specific epithet *chinensis* is assigned to reflect the species’ current distribution, which is exclusively recorded in southeastern China yet exhibits broad occupancy across this region.

### ﻿Genus Clethrorasa

Taxon classificationAnimaliaLepidopteraNoctuidae

Hampson, 1908

2937B659-4FF8-5503-A2C1-FFC16A3E1580

Clethrorasa Hampson, 1908, “Cat. Lepid. Phalaenae Br. Mus.” 7: 15 [key]; Hampson 1910, “Cat. Lepid. Phalaenae Br. Mus.” 9: 343. Type species: “Leocymapilcheri” Hampson, 1896, by original designation.

#### Description.

**Adult.** Wingspan 29.0–38.5 mm. The color of head, dorsal side of thorax, patagia, tegulae, and forewings nearly white. Antennae black-brown and linear. Proboscis well developed, with the labial palpus extending upward. Forehead smooth and devoid of protrusions. Compound eyes large. Two pairs of prominent black spots located in disc area of thorax, patagia with pair of black spots; each side of metathorax displaying a cluster of radiating white hairs. Abdomen mostly black; several segments of abdominal base adorned with brushes on dorsal side; anal tuft white, extending straight out from the posterior end of abdomen; tufts of hair arranged on lateral sides of abdomen white at distal half. Legs generally black, with white ring at each segment on the base of tarsus. Forewings slightly narrow, with scattered black, metallic blue glossy dots, blocks, or stripes. Hindwings broad, grayish-brown or partially light brown. ***Male genitalia*.** Uncus long with a simple structure; tegumen relatively wide; valva regular, with slight protrusions and absent harpe; vinculum slender; saccus shaped like a “V”; aedeagus cylindrical, featuring a tie-shaped vesica and moderately abundant cornuti. ***Female genitalia*.** Papillae anales nail-shaped and slightly flattened; anterior and posterior apophysis processes relatively slender; ductus bursae short and simple; corpus bursae relatively regular in shape, with longitudinal folds and no appendix bursae.

#### Distribution.

**Figure 22.**
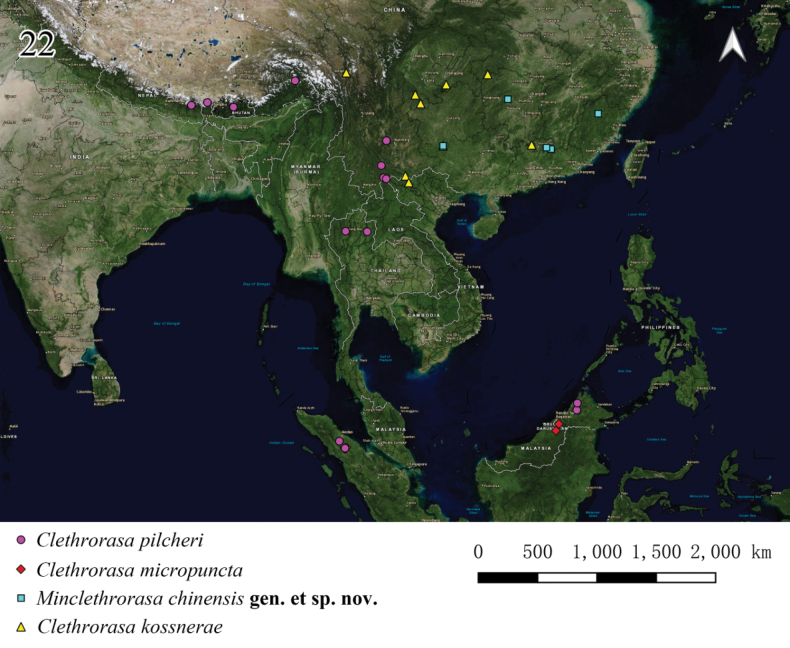
Distribution map of *Minclethrorasa* and *Clethrorasa* spp.

China, India, Nepal, Bhutan, Vietnam, Thailand, Malaysia, Indonesia, Brunei (Fig. 22).

### ﻿ Clethrorasa kossnerae

Taxon classificationAnimaliaLepidopteraNoctuidae

Behounek, 1997

E261C19C-2F1A-5CFD-B423-B3549F720676

Figs 3 , 4 , 10 , 14 , 16 , 17–18 , 22 , 24 , Table 2 Chinese common name 安南飘夜蛾 (Vietnamese Fluttering Noctuid)

Clethrorasa kossnerae Behounek, 1997, “Spixiana” 20 (3): 281, abb. 1–3, 7. Type locality: N. Vietnam, Mt. Fan-si-Pan, W-Seite, Cha-pa (= Sapa), 1600–1800 m.
Clethrorasa tibetica Orhant, 2013, syn. nov., “Lambillionea” 113(1): 30, figs 1, 2, 11. Type locality: China, Tibet, Markam County, Yangda, Chubarong.
Clethrorasa kossnerae : Holloway 2011, “Malayan Nature J.” 63 (1–2) [note].
Clethrorasa pilcheri [misidentification]: Chen 1999, “Fauna Sinica, Insecta” vol. 16: 809, pl. 29, fig. 6; Han and Yao 2018, “Atlas of Noctuidae in Guanshan National Reserve, Jiangxi Province”: 99, pl. 29, fig. 6; Wang et al. 2018, “Moths of Guangdong Nanling National Nature Reserve Supplement”: 85, pl. 23, fig. 11.

#### Material examined.

• 2 males, 2 females, China, Chongqing, Mt. Simian, Tudiyan Scenic Spot, 31.VII–4.VIII.2019, leg. T.T. Zhao and S.C. Deng, genit. prep. HHL-7040-1, 7041-1, 7033-2 and 7036-2; • 1 female, China, Yunnan, Zhaotong, Yanjin, Tantou, 18.VI.2022, leg. J. Wu et al., genit. prep. HHL-7034-2; • 1 female, China, Yunnan, Zhaotong, Luokan, Daxi, 13.VII.2023, leg. R.T. Xu, and M.X. Han, genit. prep. HHL-7035-2; • 1 female, China, Jiangxi, Guanshan, 8.IX.2004, unknown collector, genit. prep. HHL-7037-2; • 1 female, China, Jiangxi, Guanshan Nature Reserve, 21–28.VIII.2017, leg. H.L. Han, genit. prep. HHL-7038-2; • 1 male, China, Guangxi, Longsheng, Huaping National Reserve, 10–14.VII.2021, leg. J.J. Fan amd B. Gao, genit. prep. HHL-7039-1; • 1 male, China, Yunnan, Honghe, Jinping, Ma’andi, 13–19.VIII.2023, leg. N. Zhang, genit. prep. HHL-7042-1; • 1 male, China, Guangdong, Nanling, 28.VII.2008, leg. N. Zhang, genit. prep. HHL-7043-1; • 1 male, China, Guangdong, Nanling, 7–9.V.2011, leg. H.L. Han and Y.Q. Hu, genit. prep. HHL-7044-1; • 1 male, China, Hunan, Bada gong Mountain, Tianping Mountain, 2020.28 VI–27 VII 2020, leg. C.Q. Liao et al., genit. prep. HHL-7045-1.

#### Remarks.

This species was described in 1997, with a female paratype from Guangdong, China. Orhant (2013) described the species *C.tibetica* (Figs 16, 17) as a new species based on a female specimen from Markam (= Mangkang) County in SE Xizang, China. He mentioned that the report of *C.pilcheri* in Hunan by Chen (1999) should actually be classified as *C.tibetica*, although the original text did not make a comparison with *C.kossnerae*. Upon examining specimens from various provinces in southern China (including Guangdong and Hunan, extending as far west as Yunnan) and finding no significant differences in appearance and genitalia, it appears that *C.kossnerae* is widely distributed in southern China and is indeed the same as *C.tibetica*. While no specimens of similar species from Xizang were examined, a comparison of the type photographs and the original description revealed no noticeable differences in appearance and female genitalia, nor geographical isolation in distribution between *C.kossnerae* and *C.tibetica*. Therefore, based on the aforementioned considerations, the latter is deemed a junior synonym of the former. It is noteworthy that *C.kossnerae* is often misidentified as *C.pilcheri*, but the two can be distinguished by the characteristics shown in Table 2.

**Table 2.** Important characteristics to distinguish *C.kossnerae* and *C.pilcheri*.

Characteristics	C.kossnerae	C.pilcheri
**markings on the apical area of the forewings**	one L-shaped black spot (sometimes split into 2 triangular spots) and one wedge-shaped black spot	one triangular black spot with at most one additional small black spot below the outer side (sometimes merged into one spot)
**veins of the hindwings**	veins M3 and Cu1 relatively short, close to 1/3 of the hindwing length	veins M3 and Cu1 relatively long, close to 1/2 of the hindwing length
**male genitalia**	more cornuti on the vesica	less cornuti on the vesica
**female genitalia**	longer antrum on the ductus bursae	shorter antrum on the ductus bursae

#### Distribution.

China (Hunan, Jiangxi, Guangdong, Guangxi, Chongqing, Yunnan, Xizang); Vietnam.

### ﻿ Clethrorasa micropuncta

Taxon classificationAnimaliaLepidopteraNoctuidae

Holloway, 1989

E1B0F920-BEEE-5606-A414-ADF9083B17F0

Figs 5 , 6 , 11 , 22

Clethrorasa micropuncta Holloway, 1989, “Malayan Nature J.” 42 (2–3): 153, pl. 6; figs 4, 247. Type locality: Brunei, 300 m, Ulu Temburong.
Clethrorasa micropuncta : Behounek 1997, “Spixiana” 20 (3): 283, abb. 6; Holloway 2011, “Malayan Nature J.” 63 (1–2) [checklist].

#### Remarks.

According to the original description by Holloway (1989), the holotype specimen of this species originates from Temburong, Brunei, while the paratype specimens are from Brunei and Sarawak, Malaysia. However, the distribution area is documented as Borneo and Sumatra in the geographical distribution section. Based on specimen information, Behounek (1997) was the first to report the distribution in Sumatra, Indonesia. The female genitalia of this species have not been described yet.

#### Distribution.

Brunei, Malaysia (Sarawak), Indonesia (Sumatra).

### ﻿ Clethrorasa pilcheri

Taxon classificationAnimaliaLepidopteraNoctuidae

(Hampson, 1896)

70ECD549-4090-5732-AE8C-8B9F4A57B7F2

Figures 7 , 8 , 12 , 15 , 19–21 , 22 , 25 , Table 2 Chinese common name 飘夜蛾 (Fluttering Noctuid)

Leocyma pilcheri Hampson, 1896, “Fauna Br. India (Moths)” 4: 512. Type locality: Sikkim.
Clethrorasa pilcheri : Hampson 1910, “Cat. Lepid. Phalaenae Br. Mus.” 9: 343, fig. 157; Warren 1913, in Seitz, “Macrolep. World” 11: 191; Holloway 1976, “Moths of Borneo Kinabalu”: 15; Holloway 1989, “Malayan Nature J.” 42 (2–3): 153, pl. 6, figs 248, 249; Chen, 1992, in Peng, “Icon. For. Ins. Hunan”: 989, fig. 3436 [misidentification?]; Yoshimoto 1995, “Tinea” 14 (suppl. 2): 71, pl. 113, fig. 27; Behounek 1997, “Spixiana” 20 (3): 282, abb. 4–5; Holloway 2011, “Malayan Nature J.” 63 (1–2) [checklist]; Kononenko and Pinratana 2013, “Moths of Thailand” Vol. 3 (Part 2): 288, pl. 39, figs 20, 21; Orhant 2013, “Lambillionea” 113(1): figs 3, 4; Gielis et al. 2022, “Moths of Bhutan”: 129, pl. 166.

#### Material examined.

• 1 male, China, Xizang, Motuo (= Mêdog), De’ergong, 26.V–4.VI.2021, leg. H.L. Han, genit. prep. QY-1; • 1 female, China, Yunnan, Mojiang, 18–19.IX.2008, leg. H.L. Han and Y. Wang, genit. prep. HHL-7026-2; • 1 male, China, Yunnan, Jiangcheng, 15–17.IX.2008, leg. H.L Han. and M.J. Qi, genit. prep. HHL-7027-11; • 1 male, China, Yunnan, Chuxiong, Lufeng, Shimen, 29.VI.2022, W.Y. Liu et al., genit. prep. HHL-7028-1; • 1 male, Malaysia, Borneo, Mt. Trusmadi, Jungle Girl Camp, 20.IV–2.V.2016, H.L. Han, genit. prep. HHL-7029-1; • 1 male, dito, 20–25.VIll.2016, H.L. Han, genit. prep. HHL-7031-1; • 1 male, ditto, 1–6.X.2018, H.L. Han, genit. prep. HHL-7030-1; • 1 female, China, Yunnan, Lvchun, Mt. Huanglian, 27–31.VII.2018, leg. H.L. Han and J. Wu, genit. prep. HHL-7032-2.

**Figures 23–25.**
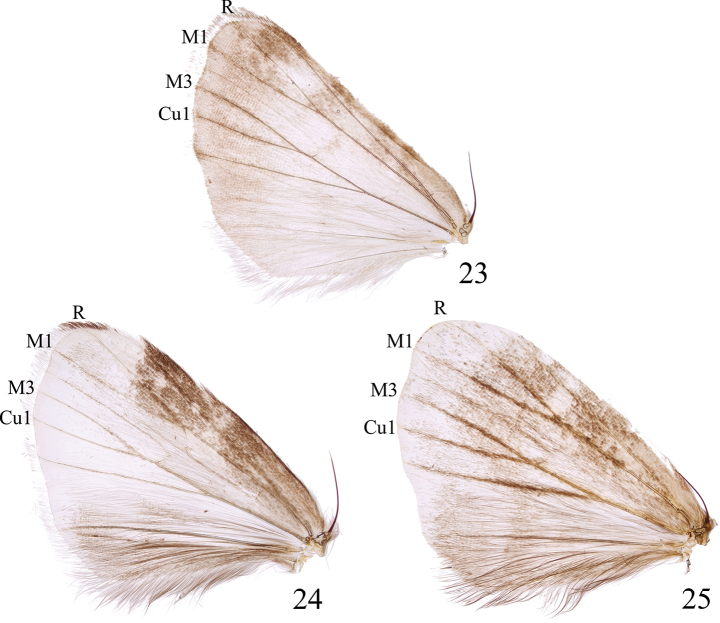
Hindwings of *Minclethrorasa* and *Clethrorasa* spp.: 23. *M.chinensis* gen. et sp. nov.; 24. *C.kossnerae* Behounek, 1997; 25. *C.pilcheri* (Hampson, 1896).

#### Supplementary description.

Female genitalia (Fig. 15). Papillae anales broad and thick, nail-shaped. Apophyses anteriores thick, while the apophyses posteriores thin and approximately equal in length. Ostium bursae relatively straight. Ductus bursae slightly short, with thick anterior half. Antrum cylindrical, strongly sclerotized, extending to membranous and spirally twisted posterior end. Corpus bursae slender, slightly curved, and tie-shaped, covered with fine folds.

#### Remarks.

This species was initially described in Sikkim (now a state in India) and later discovered in the Malay Archipelago and mainland Southeast Asia. Its distribution is the broadest among the four species in the genus, ranging from the northeastern Himalayas to Sumatra and Borneo. Compared to other species, *C.pilcheri* displays a wider variation in appearance with regionally specific phenotypes. This is mainly seen in the presence or absence of certain small spots (Figs 19, 20, 21): specimens from the Himalayan region (India: Sikkim, China: Xizang, Nepal and Bhutan), have a black-dot orbicular spot; one subbasal line; and two or three black spots near the inner margin of the forewing subterminal line. In specimens from mainland Southeast Asia (China: Yunnan, Thailand), these black spots are often absent. In specimens from the Malay Archipelago, only the black spots on the subbasal line near the inner margin are present. However, specimens collected from Xizang, Yunnan, and Borneo have only slight differences in genitalia, particularly in the smooth, small cornuti row located at the posterior part of the dorsal side of the vesica, which is a distinctive feature of the species. The variations in appearance are not significant enough to warrant interspecific differentiation. Therefore, in this article, they are considered geographical variants of the same species, and their relationship needs further clarification in conjunction with molecular data.

#### Distribution.

China (Yunnan, Xizang); India, Nepal, Bhutan, Thailand, Malaysia, Indonesia.

### ﻿Key to species of genera *Minclethrorasa* gen. nov. and *Clethrorasa* Hampson, 1908

**Table:** 

1	Dorsal surface of abdomen off-white	***Minclethrorasachinensis* gen. et sp. nov.**
−	Dorsal surface of abdomen almost completely black	**2**
2	Forewings with only one black spot near apical angle near costal margin, at most one black spot extended obliquely below the black spot	***Clethrorasapilcheri* Hampson**
−	Forewings with at least two black spots near apical angle near costal margin	**3**
3	Orbicular spot a black dot	***Clethrorasakossnerae* Behounek**
−	Orbicular spot indistinct	***Clethrorasamicropuncta* Holloway**

## Supplementary Material

XML Treatment for Minclethrorasa

XML Treatment for Minclethrorasa chinensis

XML Treatment for Clethrorasa

XML Treatment for Clethrorasa kossnerae

XML Treatment for Clethrorasa micropuncta

XML Treatment for Clethrorasa pilcheri

## ﻿Additional information

### Use of AI

No use of AI was reported.

### Author ORCIDs

Yue Qin https://orcid.org/0009-0009-3399-8560

Jiang Zhu https://orcid.org/0009-0006-1117-5449

Huilin Han https://orcid.org/0000-0002-2045-6182

### Data availability

All of the data that support the findings of this study are available in the main text.
